# (*Z*)-3-(1-Hy­droxy-3-oxobut-1-en­yl)-6-nitro-2*H*-chromen-2-one

**DOI:** 10.1107/S1600536812051872

**Published:** 2013-01-23

**Authors:** Nishith Saurav Topno, Venkataswamy Tangeti, H. Surya Prakash Rao, R. Krishna

**Affiliations:** a Centre for Bioinformatics, School of Life Sciences, Pondicherry University, Puducherry 605 014, India; b Department of Chemistry, Pondicherry University, Puducherry 605 014, India

## Abstract

In the title compound, C_13_H_9_NO_6_, the coumarin system has the benzene ring aligned at 0.61 (18)° with respect to the pyrone ring. An intra­molecular O—H⋯O hydrogen bond stabilizes the mol­ecular conformation and a C—H⋯O contact also occurs. In the crystal, weak C—H⋯O inter­actions link the mol­ecules, forming inversion dimers.

## Related literature

For the biological importance of flavinoids and coumarins, see: Murry *et al.* (1982 ▶); Andersen *et al.* (2006 ▶); Murakami *et al.* (2001 ▶); Wu *et al.* (2003 ▶). For their use as fluorescent probes and triplet sensitisers, see: Wagner (2009 ▶); Takadate *et al.* (1995 ▶). For a related structure, see: Da & Quan (2010 ▶).
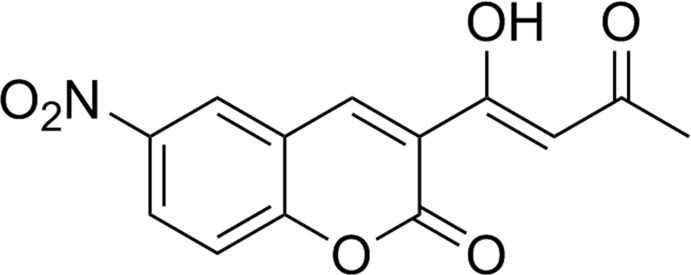


## Experimental

### 

#### Crystal data

C_13_H_9_NO_6_
*M* *_r_* = 275.21
Triclinic, 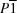

*a* = 7.4591 (13) Å
*b* = 8.2178 (19) Å
*c* = 10.0087 (18) Å
α = 85.202 (17)°
β = 77.346 (15)°
γ = 89.278 (17)°
*V* = 596.5 (2) Å^3^
*Z* = 2
Mo *K*α radiation
μ = 0.12 mm^−1^
*T* = 293 K
0.4 × 0.32 × 0.2 mm

#### Data collection

Oxford Diffraction Xcalibur Eos diffractometer
Absorption correction: multi-scan (*CrysAlis PRO*; Oxford Diffraction, 2009 ▶) *T*_min_ = 0.917, *T*_max_ = 1.000
4789 measured reflections
2093 independent reflections
1395 reflections with *I* > 2σ(*I*)
*R*_int_ = 0.033

#### Refinement

*R*[*F* ^2^ > 2σ(*F* ^2^)] = 0.048
*wR*(*F* ^2^) = 0.170
*S* = 0.93
2093 reflections
183 parameters
H-atom parameters constrained
Δρ_max_ = 0.18 e Å^−3^
Δρ_min_ = −0.20 e Å^−3^

### 

Data collection: *CrysAlis PRO* (Oxford Diffraction, 2009 ▶); cell refinement: *CrysAlis PRO*; data reduction: *CrysAlis PRO*; program(s) used to solve structure: *SHELXS97* (Sheldrick, 2008 ▶); program(s) used to refine structure: *SHELXL97* (Sheldrick, 2008 ▶); molecular graphics: *ORTEP-3 for Windows* (Farrugia, 2012 ▶); software used to prepare material for publication: *PLATON* (Spek, 2009 ▶).

## Supplementary Material

Crystal structure: contains datablock(s) I, global. DOI: 10.1107/S1600536812051872/sj5289sup1.cif

Structure factors: contains datablock(s) I. DOI: 10.1107/S1600536812051872/sj5289Isup2.hkl

Supplementary material file. DOI: 10.1107/S1600536812051872/sj5289Isup3.cml

Additional supplementary materials:  crystallographic information; 3D view; checkCIF report

## Figures and Tables

**Table 1:** Hydrogen-bond geometry (Å, °)

*D*—H⋯*A*	*D*—H	H⋯*A*	*D*⋯*A*	*D*—H⋯*A*
O3—H3*A*⋯O4	0.82	1.78	2.510 (2)	147
C11—H11⋯O2	0.93	2.24	2.870 (3)	125
C3—H3⋯O5^i^	0.93	2.58	3.308 (3)	136
C7—H7⋯O4^ii^	0.93	2.39	3.304 (3)	166

Symmetry codes: (i) 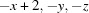; (ii) 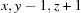.

**Fig. 1.**
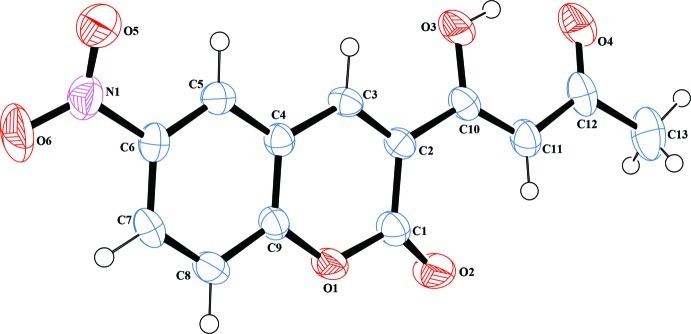
The molecular structure of (I), showing the atom-numbering scheme with displacement ellipsoids drawn at the 50% probability level.

**Fig. 2.**
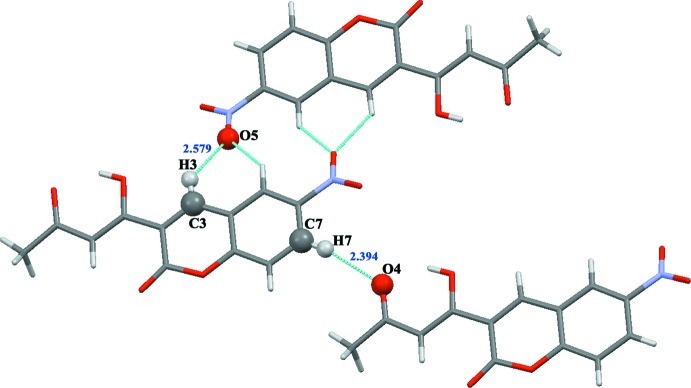
View showing the weak C–H···O intermolecular interactions in compound (I).

**Fig. 3.**
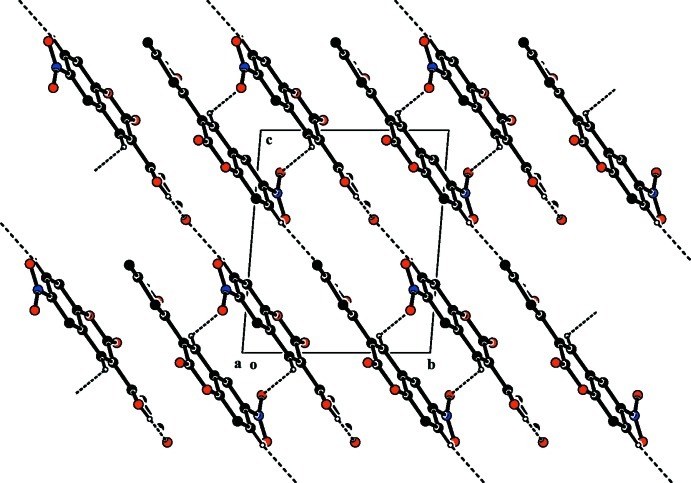
Packing diagram of the title compound (I).

## supplementary crystallographic information

### Comment

Coumarins are heterocyclic compounds belonging to the benzopyrone chemical class, well known to exhibit varied biological activities (Murry *et al.*, 1982; Andersen *et al.*, 2006). In the technological and medicinal fields, coumarins and flavones, independently, find extensive use (Murakami *et al.*, 2001) (Wu *et al.*, 2003), with activities reported for anti-HIV, anti-tumor, anti-cancer, anti-hypertension, anti-arrhythmia, anti-inflammatory, anti-osteoporosis, antiseptic, and analgesic uses. They are also known to be used as fluorescent probes and as triplet sensitizers, especially those having electronic push-pull characteristics (Wagner, 2009; Takadate *et al.*, 1995). Considering the importance of coumarin derivatives, we report here the structure of the title compound. A structure related to the title compound has also been reported (Da & Quan, 2010).

The molecular structure of the title compound is shown in Fig.1. The pyrone ring and the benzene ring are essentially co-planar with a dihedral angle of 0.61 (18)° between them. The benzene ring orients in a (-)-anti-periplanar conformation with respect to the pyrone ring. The crystal packing is stabilized by intermolecular C_3_—H_3_···O_5_, C_3_—H_3_···O_5_ and C_7_—H_7_···O_4_ bonds as shown in Fig.2 and Fig.3.

### Experimental

A solution of 4-hydroxy-6-methyl-3-(2-(methylamino)-3,6-dinitro-4*H*-chromen-4-yl)-2*H*-pyran-2-one (0.010 g, 0.266 mmol) in ethanol (15 ml) was heated to reflux for 25 min by which time the reaction was complete (TLC; hexanes: EtOAc, 6:4). The compound was crystallized and separated by filtration with the help of cold ethanol (5 ml) to yield 93% of the product, a yellow crystalline solid, mp 121.6 °C; IR (KBr) υ_max_ cm^-1^; ^1^H NMR (400 MHz, DMSO-D_6_) δ 15.71 (s, 1H), 8.70 (s, 1H), 8.58 (s, 1H), 8.49 (d, *J* = 9.0 Hz, 1H), 7.51 (d, *J* = 9.12 Hz, 1H), 6.98 (s, 1H), 2.29 (s, 3H) p.p.m.; ^13^C NMR (100 MHz, DMSO-D_6_) δ 200.2, 171.1, 157.5, 153.3, 144.0, 136.6, 131.6, 121.8, 120.1, 118.4, 117.6, 102.0, 27.8 p.p.m..

### Refinement

All hydrogen atoms were placed in calculated positions, with C—H = 0.93Å for aromatic and 0.96Å for methyl and 0.82Å for hydroxyl H atoms and were included in the refinement using a riding model with *U*_iso_(H) = x *U*_eq_(C/O), where x = 1.5 for methyl and OH and 1.2 for all other atoms.

### Crystal data

**Table:**

C_13_H_9_NO_6_	*Z* = 2
*M**_r_* = 275.21	*F*(000) = 284
Triclinic, *P*1	*D*_x_ = 1.532 Mg m^−^^3^
Hall symbol: -P 1	Mo *K*α radiation, λ = 0.71073 Å
*a* = 7.4591 (13) Å	Cell parameters from 2257 reflections
*b* = 8.2178 (19) Å	θ = 3.1–29.1°
*c* = 10.0087 (18) Å	µ = 0.12 mm^−^^1^
α = 85.202 (17)°	*T* = 293 K
β = 77.346 (15)°	Plate, colorless
γ = 89.278 (17)°	0.4 × 0.32 × 0.2 mm
*V* = 596.5 (2) Å^3^	

### Data collection

**Table:**

Oxford Diffraction Xcalibur Eos diffractometer	2093 independent reflections
Radiation source: fine-focus sealed tube	1395 reflections with *I* > 2σ(*I*)
Graphite monochromator	*R*_int_ = 0.033
Detector resolution: 15.9821 pixels mm^-1^	θ_max_ = 25.0°, θ_min_ = 3.1°
ω scans	*h* = −8→8
Absorption correction: multi-scan (*CrysAlis PRO*; Oxford Diffraction, 2009)	*k* = −9→8
*T*_min_ = 0.917, *T*_max_ = 1.000	*l* = −11→11
4789 measured reflections	

### Refinement

**Table:**

Refinement on *F*^2^	Primary atom site location: structure-invariant direct methods
Least-squares matrix: full	Secondary atom site location: difference Fourier map
*R*[*F*^2^ > 2σ(*F*^2^)] = 0.048	Hydrogen site location: inferred from neighbouring sites
*wR*(*F*^2^) = 0.170	H-atom parameters constrained
*S* = 0.93	*w* = 1/[σ^2^(*F*_o_^2^) + (0.1*P*)^2^] where *P* = (*F*_o_^2^ + 2*F*_c_^2^)/3
2093 reflections	(Δ/σ)_max_ < 0.001
183 parameters	Δρ_max_ = 0.18 e Å^−^^3^
0 restraints	Δρ_min_ = −0.20 e Å^−^^3^

### Special details

**Table:**

Geometry. All e.s.d.'s (except the e.s.d. in the dihedral angle between two l.s. planes) are estimated using the full covariance matrix. The cell e.s.d.'s are taken into account individually in the estimation of e.s.d.'s in distances, angles and torsion angles; correlations between e.s.d.'s in cell parameters are only used when they are defined by crystal symmetry. An approximate (isotropic) treatment of cell e.s.d.'s is used for estimating e.s.d.'s involving l.s. planes.
Refinement. Refinement of *F*^2^ against ALL reflections. The weighted *R*-factor *wR* and goodness of fit *S* are based on *F*^2^, conventional *R*-factors *R* are based on *F*, with *F* set to zero for negative *F*^2^. The threshold expression of *F*^2^ > σ(*F*^2^) is used only for calculating *R*-factors(gt) *etc*. and is not relevant to the choice of reflections for refinement. *R*-factors based on *F*^2^ are statistically about twice as large as those based on *F*, and *R*- factors based on ALL data will be even larger.

### Fractional atomic coordinates and isotropic or equivalent isotropic displacement parameters (Å^2^)

**Table:**

	*x*	*y*	*z*	*U*_iso_*/*U*_eq_	
O1	0.25449 (19)	0.17075 (18)	0.16604 (16)	0.0422 (5)	
C4	0.5804 (3)	0.1391 (2)	0.1024 (2)	0.0306 (5)	
C2	0.4261 (3)	0.3162 (2)	−0.0432 (2)	0.0318 (5)	
C5	0.7376 (3)	0.0665 (2)	0.1335 (2)	0.0350 (6)	
H5	0.8525	0.0887	0.0766	0.042*	
C3	0.5812 (3)	0.2510 (2)	−0.0161 (2)	0.0329 (5)	
H3	0.6927	0.2791	−0.0757	0.040*	
C9	0.4119 (3)	0.1020 (3)	0.1911 (2)	0.0337 (5)	
O3	0.6017 (2)	0.4703 (2)	−0.23192 (17)	0.0497 (5)	
H3A	0.5984	0.5364	−0.2974	0.075*	
O4	0.4619 (3)	0.6406 (2)	−0.40237 (18)	0.0584 (6)	
C11	0.2837 (3)	0.4849 (3)	−0.2152 (2)	0.0417 (6)	
H11	0.1660	0.4551	−0.1668	0.050*	
C6	0.7191 (3)	−0.0382 (3)	0.2494 (2)	0.0367 (6)	
C8	0.3959 (3)	−0.0051 (3)	0.3076 (2)	0.0402 (6)	
H8	0.2815	−0.0284	0.3648	0.048*	
O2	0.1007 (2)	0.3272 (2)	0.04475 (19)	0.0620 (6)	
C10	0.4319 (3)	0.4287 (2)	−0.1671 (2)	0.0344 (6)	
N1	0.8848 (3)	−0.1149 (2)	0.2826 (2)	0.0474 (6)	
C7	0.5514 (3)	−0.0762 (3)	0.3373 (2)	0.0393 (6)	
H7	0.5444	−0.1485	0.4149	0.047*	
C1	0.2494 (3)	0.2775 (3)	0.0523 (2)	0.0394 (6)	
O6	0.8773 (3)	−0.1692 (3)	0.3998 (2)	0.0900 (8)	
O5	1.0203 (2)	−0.1232 (2)	0.1907 (2)	0.0639 (6)	
C13	0.1405 (4)	0.6402 (4)	−0.3939 (3)	0.0728 (9)	
H13A	0.1784	0.6891	−0.4863	0.109*	
H13B	0.0716	0.7180	−0.3369	0.109*	
H13C	0.0651	0.5462	−0.3932	0.109*	
C12	0.3071 (4)	0.5893 (3)	−0.3397 (3)	0.0474 (6)	

### Atomic displacement parameters (Å^2^)

**Table:**

	*U*^11^	*U*^22^	*U*^33^	*U*^12^	*U*^13^	*U*^23^
O1	0.0340 (9)	0.0494 (10)	0.0382 (10)	0.0047 (7)	−0.0036 (7)	0.0134 (7)
C4	0.0345 (12)	0.0279 (12)	0.0291 (12)	−0.0022 (8)	−0.0071 (9)	0.0012 (8)
C2	0.0367 (12)	0.0279 (12)	0.0297 (13)	−0.0012 (9)	−0.0064 (10)	0.0017 (9)
C5	0.0315 (12)	0.0373 (13)	0.0348 (13)	−0.0012 (9)	−0.0054 (10)	0.0014 (10)
C3	0.0332 (12)	0.0324 (12)	0.0302 (12)	−0.0031 (9)	−0.0022 (9)	0.0024 (9)
C9	0.0344 (12)	0.0354 (12)	0.0308 (12)	0.0018 (9)	−0.0076 (9)	0.0013 (9)
O3	0.0494 (11)	0.0562 (12)	0.0381 (11)	−0.0036 (8)	−0.0053 (8)	0.0177 (8)
O4	0.0770 (13)	0.0554 (12)	0.0395 (11)	−0.0030 (9)	−0.0123 (9)	0.0143 (8)
C11	0.0491 (15)	0.0397 (14)	0.0365 (14)	0.0011 (10)	−0.0135 (11)	0.0062 (10)
C6	0.0406 (13)	0.0361 (13)	0.0363 (13)	0.0046 (9)	−0.0156 (10)	−0.0016 (9)
C8	0.0383 (13)	0.0486 (14)	0.0295 (13)	−0.0025 (10)	−0.0022 (10)	0.0068 (10)
O2	0.0348 (10)	0.0816 (14)	0.0625 (13)	0.0106 (8)	−0.0080 (8)	0.0261 (10)
C10	0.0436 (13)	0.0297 (12)	0.0287 (12)	−0.0019 (9)	−0.0059 (10)	0.0003 (9)
N1	0.0478 (13)	0.0527 (13)	0.0445 (13)	0.0056 (9)	−0.0198 (10)	0.0041 (10)
C7	0.0482 (14)	0.0403 (13)	0.0283 (13)	0.0022 (10)	−0.0089 (10)	0.0049 (9)
C1	0.0409 (13)	0.0405 (14)	0.0342 (13)	0.0030 (10)	−0.0066 (10)	0.0065 (10)
O6	0.0764 (15)	0.142 (2)	0.0506 (13)	0.0326 (14)	−0.0257 (11)	0.0264 (13)
O5	0.0431 (11)	0.0806 (14)	0.0632 (14)	0.0153 (9)	−0.0091 (9)	0.0125 (10)
C13	0.090 (2)	0.078 (2)	0.0564 (19)	0.0096 (16)	−0.0389 (17)	0.0148 (15)
C12	0.0694 (18)	0.0401 (14)	0.0362 (14)	0.0019 (12)	−0.0209 (13)	0.0022 (11)

### Geometric parameters (Å, º)

**Table:**

O1—C9	1.360 (3)	C11—C12	1.432 (3)
O1—C1	1.385 (3)	C11—H11	0.9300
C4—C9	1.391 (3)	C6—C7	1.384 (3)
C4—C5	1.392 (3)	C6—N1	1.470 (3)
C4—C3	1.438 (3)	C8—C7	1.370 (3)
C2—C3	1.341 (3)	C8—H8	0.9300
C2—C1	1.470 (3)	O2—O2	0.0000
C2—C10	1.476 (3)	O2—C1	1.192 (3)
C5—C6	1.368 (3)	N1—O6	1.210 (3)
C5—H5	0.9300	N1—O5	1.214 (2)
C3—H3	0.9300	C7—H7	0.9300
C9—C8	1.385 (3)	C1—O2	1.192 (3)
O3—C10	1.325 (3)	C13—C12	1.502 (4)
O3—H3A	0.8200	C13—H13A	0.9600
O4—O4	0.000 (5)	C13—H13B	0.9600
O4—C12	1.247 (3)	C13—H13C	0.9600
C11—C10	1.361 (3)	C12—O4	1.247 (3)
			
C9—O1—C1	123.35 (17)	O3—C10—C11	121.5 (2)
C9—C4—C5	118.4 (2)	O3—C10—C2	112.69 (18)
C9—C4—C3	117.68 (19)	C11—C10—C2	125.8 (2)
C5—C4—C3	123.93 (19)	O6—N1—O5	123.5 (2)
C3—C2—C1	119.73 (19)	O6—N1—C6	118.0 (2)
C3—C2—C10	120.49 (19)	O5—N1—C6	118.4 (2)
C1—C2—C10	119.77 (18)	C8—C7—C6	118.7 (2)
C6—C5—C4	118.5 (2)	C8—C7—H7	120.7
C6—C5—H5	120.8	C6—C7—H7	120.7
C4—C5—H5	120.8	O2—C1—O2	0.00 (18)
C2—C3—C4	121.97 (19)	O2—C1—O1	115.3 (2)
C2—C3—H3	119.0	O2—C1—O1	115.3 (2)
C4—C3—H3	119.0	O2—C1—C2	128.1 (2)
O1—C9—C8	117.09 (19)	O2—C1—C2	128.1 (2)
O1—C9—C4	120.68 (19)	O1—C1—C2	116.55 (18)
C8—C9—C4	122.2 (2)	C12—C13—H13A	109.5
C10—O3—H3A	109.5	C12—C13—H13B	109.5
O4—O4—C12	0 (10)	H13A—C13—H13B	109.5
C10—C11—C12	120.7 (2)	C12—C13—H13C	109.5
C10—C11—H11	119.7	H13A—C13—H13C	109.5
C12—C11—H11	119.7	H13B—C13—H13C	109.5
C5—C6—C7	123.2 (2)	O4—C12—O4	0.0 (2)
C5—C6—N1	118.6 (2)	O4—C12—C11	121.4 (2)
C7—C6—N1	118.2 (2)	O4—C12—C11	121.4 (2)
C7—C8—C9	119.0 (2)	O4—C12—C13	119.6 (2)
C7—C8—H8	120.5	O4—C12—C13	119.6 (2)
C9—C8—H8	120.5	C11—C12—C13	119.0 (3)
O2—O2—C1	0 (10)		
			
C9—C4—C5—C6	0.2 (3)	C7—C6—N1—O6	−20.2 (4)
C3—C4—C5—C6	180.0 (2)	C5—C6—N1—O5	−21.4 (3)
C1—C2—C3—C4	2.1 (3)	C7—C6—N1—O5	158.3 (2)
C10—C2—C3—C4	−179.02 (19)	C9—C8—C7—C6	0.0 (4)
C9—C4—C3—C2	−1.2 (3)	C5—C6—C7—C8	−0.4 (4)
C5—C4—C3—C2	178.94 (19)	N1—C6—C7—C8	179.8 (2)
C1—O1—C9—C8	−178.8 (2)	O2—O2—C1—O1	0.00 (3)
C1—O1—C9—C4	1.2 (3)	O2—O2—C1—C2	0.00 (10)
C5—C4—C9—O1	179.40 (19)	C9—O1—C1—O2	179.87 (19)
C3—C4—C9—O1	−0.4 (3)	C9—O1—C1—O2	179.87 (19)
C5—C4—C9—C8	−0.6 (3)	C9—O1—C1—C2	−0.4 (3)
C3—C4—C9—C8	179.6 (2)	C3—C2—C1—O2	178.4 (2)
C4—C5—C6—C7	0.3 (4)	C10—C2—C1—O2	−0.5 (4)
C4—C5—C6—N1	−179.93 (18)	C3—C2—C1—O2	178.4 (2)
O1—C9—C8—C7	−179.48 (18)	C10—C2—C1—O2	−0.5 (4)
C4—C9—C8—C7	0.5 (4)	C3—C2—C1—O1	−1.2 (3)
C12—C11—C10—O3	1.0 (4)	C10—C2—C1—O1	179.84 (19)
C12—C11—C10—C2	−177.7 (2)	O4—O4—C12—C11	0.00 (14)
C3—C2—C10—O3	−8.2 (3)	O4—O4—C12—C13	0.00 (10)
C1—C2—C10—O3	170.69 (19)	C10—C11—C12—O4	−4.9 (4)
C3—C2—C10—C11	170.6 (2)	C10—C11—C12—O4	−4.9 (4)
C1—C2—C10—C11	−10.5 (3)	C10—C11—C12—C13	176.3 (2)
C5—C6—N1—O6	160.0 (2)		

### Hydrogen-bond geometry (Å, º)

**Table:**

*D*—H···*A*	*D*—H	H···*A*	*D*···*A*	*D*—H···*A*
O3—H3*A*···O4	0.82	1.78	2.510 (2)	147
C11—H11···O2	0.93	2.24	2.870 (3)	125
C3—H3···O5^i^	0.93	2.58	3.308 (3)	136
C7—H7···O4^ii^	0.93	2.39	3.304 (3)	166

Symmetry codes: (i) −*x*+2, −*y*, −*z*; (ii) *x*, *y*−1, *z*+1.
